# Editorial: Fundamental and practical advances in bioremediation of emerging pollutants as add-on treatments for polluted waters

**DOI:** 10.3389/fmicb.2024.1514698

**Published:** 2024-12-11

**Authors:** Francis Hassard, Victor Castro-Gutierrez

**Affiliations:** ^1^Cranfield University, Bedford, United Kingdom; ^2^Environmental Pollution Research Center, University of Costa Rica, San José, Costa Rica

**Keywords:** bioremediation, soil, water, nature based solutions, bioaugmentation, biofilm

Access to clean water is a fundamental human right and essential for sustainable development. Freshwater resources are increasingly strained due to industrialization, urbanization, and intensive agriculture introducing emerging contaminants into aquatic ecosystems (Ayilara and Babalola, 2023). Xenobiotic pollutants, including pesticides, pharmaceuticals, microplastics, and “forever chemicals”—threaten environmental integrity and pose risks to human health (Štefanac et al., 2021), directly impacting attainment of United Nations Sustainable Development Goals (SDGs) on clean water (SDG 6), health (SDG 3), and life below water (SDG 14).

Bioremediation offers a promising, sustainable solution by harnessing the vast metabolic potential of microorganisms, plants, and other organisms to degrade or remove harmful chemicals from the environment (Azubuike et al., 2016). However, widespread adoption has been limited by challenges in scalability, specificity, and integration with existing “black-box” treatment systems (Nandy et al., 2021). This Research Topic shows recent innovations across a wide-range of bioremediation technologies, emphasizing region-specific and tailored strategies to tackle emerging contaminants in aquatic ecosystems.

We are pleased to present a collection of articles highlighting significant progress in the field:

1. ***Biological treatment of pesticide-containing wastewater in coffee farms*** (Oviedo-Matamoros et al.): This study evaluates and optimizes biopurification systems (BPS) for Costa Rican coffee farms using biomixtures containing coconut fiber, rice husk, and coffee husk as lignocellulosic substrates. The optimized biomixtures effectively removed various pesticides, including chlorpyrifos, demonstrating a practical on-farm system that promotes sustainable agriculture and reduces environmental contamination.
2. ***Nature-based solutions for the removal of steroid oestrogens in wastewater*** (Liyanage et al.): This review explores the efficacy of treatment wetlands (TWs) and high-rate algal ponds (HRAPs) in removing natural and synthetic steroid oestrogens from wastewater in small communities. The study highlights removal efficiencies exceeding 80% and suggests alternative substrates like biochar and palm mulch to enhance performance. Innovations such as intensified multilayer wetland filters (IMWF) reduce the spatial footprint, offering practical solutions for decentralized wastewater treatment.
3. ***Bioremediation of contaminated soil and groundwater by in*** ***situ biostimulation*** (Romantschuk et al.): This review assesses *in situ* biostimulation as an effective alternative for remediating contaminated soils and groundwater by enhancing indigenous microbial communities. It highlights key bottlenecks like electron acceptor limitations and nutrient deficiencies and discusses combining biostimulation with bioaugmentation and chemical treatments to address persistent pollutants. The authors advocate for thorough site analysis, integration of sustainability assessments, and the use of AI tools to optimize remediation efforts.
4. ***Impact of carbon sources in airport de-icing compounds on the growth of Sphaerotilus natans*** (Exton et al.): This study investigates how airport de-icing compounds contribute to the growth of *Sphaerotilus natans*, a bacterium responsible for undesirable river biofilms. Using freeze-point depressants such as propylene glycol, ethylene glycol, and sodium acetate, the research shows that *S. natans* can grow on all tested compounds, highlighting ecological risks from airport runoff and informing bioremediation approaches to mitigate biofilm growth in affected waters.

While these studies show the potential of bioremediation, contaminants like per- and polyfluoroalkyl substances (PFAS) present significant challenges due to their resistance to degradation (Berhanu et al., 2023). Addressing these persistent pollutants may require advanced physico-chemical pre-treatment processes, adsorption methods, or engineered microbial solutions beyond traditional bioremediation techniques such as synthetic biology (Lu et al., 2020).

Collectively, the contributions in this Research Topic illustrate the important role of bioremediation in sustainable water management. By focusing on practical, scalable, and region-specific solutions, these studies pave the way for broader applications across various sectors. Studies show the importance of interdisciplinary collaboration, integrating microbiology, environmental engineering, and policy-making to develop effective contaminant removal strategies. As editors, we encourage the research community to continue advancing this green technology agenda. Approaches to integrate bioremediation with conventional treatment systems, scale up successful pilots, and tackle challenges posed by persistent contaminants should be prioritized. The advancements highlighted here represent small but notable strides toward that goal, bringing us closer to a sustainable future where water resources are protected and preserved. We look forward to further innovations in this vital field and are confident that a tangible impact on global water quality can be made.
